# Global Habitat Suitability of *Spodoptera frugiperda* (JE Smith) (Lepidoptera, Noctuidae): Key Parasitoids Considered for Its Biological Control

**DOI:** 10.3390/insects12040273

**Published:** 2021-03-24

**Authors:** Ghislain T. Tepa-Yotto, Henri E. Z. Tonnang, Georg Goergen, Sevgan Subramanian, Emily Kimathi, Elfatih M. Abdel-Rahman, Daniel Flø, Karl H. Thunes, Komi K. M. Fiaboe, Saliou Niassy, Anani Bruce, Samira A. Mohamed, Manuele Tamò, Sunday Ekesi, May-Guri Sæthre

**Affiliations:** 1 Biorisk Management Facility (BIMAF), International Institute of Tropical Agriculture (IITA-Benin), 08-01000 Cotonou, Benin; G.Goergen@cgiar.org (G.G.); M.Tamo@cgiar.org (M.T.); 2 Ecole de Gestion et de Production Végétale et Semencière (EGPVS), Université Nationale d’Agriculture (UNA), 43 Kétou, Benin; 3 Plant Health Theme, International Centre of Insect Physiology and Ecology (*icipe*), Nairobi 30772-00100, Kenya; htonnang@icipe.org (H.E.Z.T.); ssubramania@icipe.org (S.S.); ekimathi@icipe.org (E.K.); eabdel-rahman@icipe.org (E.M.A.-R.); Karl.Thunes@nibio.no (K.H.T.); sniassy@icipe.org (S.N.); sfaris@icipe.org (S.A.M.); sekesi@icipe.org (S.E.); 4 Norwegian Scientific Committee for Food and Environment (VKM), 0213 Oslo, Norway; Daniel.Flo@vkm.no; 5 Department for Invertebrate Pests and Weeds in Forestry, Horticulture and Agriculture, Norwegian Institute of Bioeconomy Research (NIBIO), NO-1431 Ås, Norway; 6 Department of Integrated Pest Management, International Institute of Tropical Agriculture (IITA-Cameroon), BP-2008 Messa-Yaounde, Cameroon; K.Fiaboe@cgiar.org; 7 Global Maize Program, International Maize and Wheat Improvement Center (CIMMYT), 1041-00621 Nairobi, Kenya; A.Bruce@cgiar.org; 8 Department for Climate, Energy and Environment, Section for Environment and Food Security, Norwegian Agency for Development and Cooperation (NORAD), 0257 Oslo, Norway; May-Guri.Saethre@norad.no

**Keywords:** fall armyworm, climate change, pest management, machine-learning algorithm, decision support

## Abstract

**Simple Summary:**

The fall armyworm (FAW), *Spodoptera frugiperda* has now become a pest of global importance. Its introduction and detection in Africa in 2016, and subsequent introduction and spread into Asia and Australia, has put several millions of food producers and maize farmers at risk. Not all pest management strategies are sustainable. Biological control with the use of parasitoid wasps is one of the durable and environmentally sound options. The present study was initiated to predict the habitats of high establishment potential of key parasitoids of FAW in South America, which might prove to be effective as classical biological control agents of FAW in regions where it is an invasive species under current and future climate scenarios. The prospective parasitoids are the following: *Chelonus insularis*, *Cotesia marginiventris,* *Eiphosoma laphygmae,* *Telenomus remus* and *Trichogramma pretiosum*. The results demonstrate overlapping habitat suitability areas of the pest and the parasitoids, suggesting promises for biological control options for the management of FAW under current and future climate scenarios.

**Abstract:**

The present study is the first modeling effort at a global scale to predict habitat suitability of fall armyworm (FAW), *Spodoptera frugiperda* and its key parasitoids, namely *Chelonus insularis*, *Cotesia marginiventris,* *Eiphosoma laphygmae,* *Telenomus remus* and *Trichogramma pretiosum*, to be considered for biological control. An adjusted procedure of a machine-learning algorithm, the maximum entropy (Maxent), was applied for the modeling experiments. Model predictions showed particularly high establishment potential of the five hymenopteran parasitoids in areas that are heavily affected by FAW (like the coastal belt of West Africa from Côte d’Ivoire (Ivory Coast) to Nigeria, the Congo basin to Eastern Africa, Eastern, Southern and Southeastern Asia and some portions of Eastern Australia) and those of potential invasion risks (western & southern Europe). These habitats can be priority sites for scaling FAW biocontrol efforts. In the context of global warming and the event of accidental FAW introduction, warmer parts of Europe are at high risk. The effect of winter on the survival and life cycle of the pest in Europe and other temperate regions of the world are discussed in this paper. Overall, the models provide pioneering information to guide decision making for biological-based medium and long-term management of FAW across the globe.

## 1. Introduction

Invasive species and climate change increasingly threaten the economy worldwide [1]. The continuing spread of fall armyworm (FAW), *Spodoptera frugiperda* (J E Smith) (Lepidoptera, Noctuidae) to new ecological niches raises regional and global concerns. An updated literature review and surveys recently indicated a total of 353 FAW larval host plant records belonging to 76 plant families, chiefly Poaceae (106), Asteraceae (31) and Fabaceae (31) [2]. However, the most preferred host of the pest is maize. FAW is native to tropical and subtropical regions of the Americas. The pest is known to have high dispersal capabilities. After the first report on its outbreak in Africa [3], farm areas of nearly 25 million hectares of its main host plant (maize) production have been severely compromised within only two years post detection of this pest on the continent.

FAW was first detected in Central and Western Africa in early 2016 (Benin, Nigeria, São Tomé and Príncipe, and Togo); and later reported in the whole of Southern Africa’s inland except Lesotho, in Madagascar and the Seychelles. Subsequently, it quickly spread across almost all of sub-Saharan Africa (SSA). As of July 2018, it was confirmed in India and Yemen [4]. In December 2018, FAW was reported in Bangladesh, Sri Lanka and Thailand. By June 2019, it was reported in Myanmar, China, Indonesia, Laos, Malaysia, Vietnam, Egypt and the Republic of Korea. The first report on the presence of FAW in Japan was in July 2019. In February 2020, *S. frugiperda* was officially reported in Australia and Mauritania and later in Timor-Leste in March 2020 [5]. The pest was further reported widely in Australia, Jordan and United Arab Emirates [6]. Hence, with its extensive spread, FAW has the potential to negatively impact the food security of billions of people, which calls for a collective and holistic approach for its global management [7]. Indeed, nearly all models developed to date, including this study, support potential invasion risks into the European continent [8,9,10]. In its native range Brazil, control costs of FAW are estimated to exceed 600 million US dollars (USD) per year [11]. Approximations anticipate that in the absence of appropriate control measures, FAW could risk crops valued at USD 13 billion per annum across SSA [12,13]. China is the biggest maize producer in Asia and the second-largest producer globally. Economic losses there and in other Asian countries, could be significant and severely impacting. As the distribution range of the pest is expanding worldwide, the need for designing long-term management strategies based on right decision guidance becomes crucial.

Biological control programs focus on the use of natural enemies that are host specific, effective at low densities of the pest and easy to mass-rear, handle and distribute over large areas. Therefore, the selection of parasitoids with the right attributes is key for biologically based control options that find practical applications in three main approaches: (a) introductory (classical) biological control (b) augmentative (inundative) biological control and (c) conservation biological control. In extensive inventories in the Americas, some 150 parasitoids were found to be associated with FAW [14]. Among these, *Telenomus remus* (Nixon) (Hymenoptera: Platygastridae) and *Trichogramma pretiosum* (Riley) (Hymenoptera: Trichogrammatidae) were recognized as the most relevant naturally occurring egg parasitoids. Inoculative field introductions, but particularly augmentative releases with these species, have resulted in appreciable control levels of FAW in South America [15]. In addition to egg parasitoids, the egg-larval and larval parasitoids, *Chelonus insularis* (Cresson) and *Cotesia marginiventris* (Cresson) (both Hymenoptera: Braconidae), and *Eiphosoma laphygmae* Costa Lima (Hymenoptera: Ichneumonidae), respectively, emerged among the most prevalent natural enemies, and have also proven to be efficient against FAW [15,16,17]. Beside a pest risk clearance, the selection of a candidate parasitoid species for a biological control program is based on its reproductive performance, functional and numerical response profile, high behavioral host selection, resilience at low host population densities and dispersal capabilities [18,19,20,21]. However, for a candidate parasitoid to be successful in a given ecology, environmental and climatic variables coupled to landscape management features, suitability and prevalence of the host insect and its host plants, are key determinants [22,23].

Consequently, the present work aims at modeling current global distribution and future areas at risk of the invasive fall armyworm, as well as habitat suitability of five coevolved FAW parasitoids to support decision making for releases in the newly invaded continents Africa, Asia and Australia. In this work, we examined through the maximum entropy (Maxent) algorithm, known for its high performance compared with other ecological niche models [24,25,26], how likely a decision of deploying the five parasitoid species mentioned above would cope with current and future climate scenarios in major world agroecologies or regions already threatened by, or with potential risk, of FAW invasion. The current paper discusses the potential role of biological control using parasitoids as a key component of sustainable integrated management of FAW, with reference to global future climate conditions.

## 2. Materials and Methods

### 2.1. Species Studied and Presence Records

The risks associated with the invasion of FAW were modeled using the International Institute of Tropical Agriculture (IITA)’, the International Centre of Insect Physiology and Ecology (*icipe*)’, the International Maize and Wheat Improvement Center (CIMMYT)’ and collaborators’ records across Africa. Additional FAW occurrence data were sourced from the Global Biodiversity Information Facility (GBIF, www.gbif.org (accessed 15 January 2020)) [27], the Centre for Agriculture and Biosciences International (CABI)’ Crop Protection Compendium (CPC, https://www.cabi.org/cpc/about/ (accessed on 15 January 2020)) [28] and records in India [4]. Presence records of *Te. remus* and *Ch. insularis* were scanty in these repositories. Additional points of the parasitoids were sourced through a literature review (Table S1). The records of FAW and its parasitoid *Co. marginiventris* included locations of archipelagos or islands in the North Pacific, North Atlantic, and Indian Oceans, the Hawaiian Islands, Bermuda, São Tomé and Príncipe, Cape Verde and the Seychelles. New records of *Te. remus* on the African continent [29,30] and in China [31] were mainly taken from recent reports. Presence records used for all the six modeled species are provided in Files S1-6.

### 2.2. Environmental Variables

All bioclimatic variables were sourced from WorldClim (www.worldclim.org (accessed on 15 January 2020)) [32,33]. We obtained current climate data for the period 1970–2000 at 2.5 arc minutes longitude/latitude degree spatial resolution (approximately 4.5 km at the equator). At the same resolution, the downscaled and calibrated horizon 2050 IPPC5 (CMIP5) of climate projection bioclimatic variables were extracted from two global climate models (GCMs) and for one representative concentration pathways (RCP8.5). The RCP8.5 scenario describes mean temperature increase projections of 3.7 °C.

To reduce the multicollinearity among the environmental variables, we ran Pearson correlation tests with a threshold of correlation coefficient |r| > 0.7. Variables that did not meet this criterion were removed from the models using the ‘Findcorrelation’ function in R software [34].

### 2.3. Modeling Procedures

The maximum entropy algorithm (Maxent) was used to predict global environmental suitability of *S. frugiperda* and five key parasitoids considered for its biological control, *Ch. insularis*, *Co. marginiventris*, *E. laphygmae*, *Te. remus* and *Tr. pretiosum*, respectively. Presence data used for modeling fall armyworm were divided into two sets; the northern United States of America and Canada representing the temporary migrated regions, while the other points covered the south of United States of America (Florida and Texas), and the rest of the global extent were used to represent the native and invaded regions. The data were used to develop two sets of models with two climatic scenarios. FAW is already present on all continents except Europe where it was only intercepted; therefore, temporary Northern United States of America and Canada records were used to predict habitat suitability of the pest in the event of migration to cold climates, where the species cannot survive all year-round, with a focus on European continent.

Maxent has been demonstrated to perform relatively well in the context of developing models using presence data only as input [35]. Its predictions rely on the ability to estimate a distribution of probability based on the physics science principle of “maximum entropy” that satisfies a set of checks from environmental variables. The output of Maxent is a probability viewed as the level of environmental suitability, also considered as the species ecological niche. To carry out the analysis, two GCMs from ensemble models were selected (Table 1). The first GCM used is one of the warmer CMIP5 models for almost all locations: HadGEM2-ES (4.6 °C climate sensitivity). This GCM was associated to a relatively cool model over much of the land area GISS-E2-R (2.1 °C) [36]. Accurate variables selection reduces multicollinearity among the 19 bioclimatic variables [37]. We ran initial experiments using selected bioclimatic variables (Bio1, Bio2, Bio5, Bio6, Bio9, Bio10, Bio12, Bio16 and Bio17) (Tables S2–S7) [38]. Another batch of initial experiments was run using all 19 bioclimatic variables in the models. The five most important variables with highest percent variable contributions of each of the 24 initial constellations (six species × two sets of environmental layer × two climate scenarios) were selected as described in a recent work [38] to form a reduced and consolidated set of seven environmental layers used in the final modeling stages (Tables S2–S7). The selected bioclimatic variables were well in agreement with key factors influencing insect species’ bioecology and flight activity.

### 2.4. Prediction Maps

The ecological niche model outputs of FAW, *S. frugiperda*, and five parasitoids; *Ch. insularis*, *Co. marginiventris*, *E. laphygmae*, *Te. remus* and *Tr. pretiosum*, for the current and future climate scenarios, were used to develop the species suitability maps. The Maxent probability outputs considered in this study as suitability values ranging from 0 to 1 were classified into four classes; Unsuitable (0), Low (0.1–0.3), Moderate (0.3–0.5) and high (0.5–1) using QGIS 3.10 software. To estimate the areas where the combination of the selected parasitoids could be used in classical or augmentative biological control of FAW, we combined all the five model outputs for the parasitoids, creating one raster layer showing the potential areas of suitability. This was done through a GIS raster computation (cell statistics tool) to produce an ensemble map of the model outputs from the individual models developed. The tool combines the suitability levels (0–1) for all the models of the five parasitoids species to produce a layer indicating the cumulative values ranging from 0–5. Regions with values close to 5 represent areas that the models predict to have high suitability for all the parasitoid species. On the other hand, values close to 1 represent areas suitable for only one parasitoid. The FAW field records were overlaid on the cumulative parasitoids suitability map at a global extent to identify the overlap between invasive areas and climates potentially suitable for biological control. The ensemble habitat suitability niche map using the current climate conditions was subtracted from the future RCP8.5 map to determine habitat suitability range shift.

### 2.5. Model Calibration

Presence records were used against bioclimatic variables to develop ecological niche models of *S. frugiperda* at a global extent. The first model used the native and invaded population to assess the climate suitability at a global extent. The second model utilized the temporary migrated population of the pest to model the climate suitability in the United States and Canada and then projected the model to a global extent. Seventy percent (70%) of the occurrence points were utilized to train the model, while 30% of the points were used to validate the performance of the model with the aid of MaxEnt (3.4.1) software (American Museum of Natural History, New York City, NY, USA). The models were replicated three times using a cross-validation method, and an ensemble of the three probability outputs was used to determine the optimum invasion risk and performance of the models. The comparative relevance of each environmental predictor for the models of *S. frugiperda* was evaluated using the overall percentage contribution, the area under the curve (AUC) and the Jackknife test. The ecological niche model outputs of FAW for the current and 8.5 scenarios were used to develop suitability maps. The suitability values ranging from 0 to 1 were classified into four classes: Unsuitable (0), Low (0.1–0.3), Moderate (0.3–0.5) and High (0.5–1) using QGIS 3.10.6 software (QGIS. ORG, Zürich, Switzerland).

## 3. Results

### 3.1. Evaluation of Models

The Maxent model outputs for both FAW and its parasitoids provided tight goodness of fit with an area under curve (AUC) value range of 0.802–0.971, which demonstrates that the models showed good predictive performance (Table 2 and Table 3). Annual Mean Temperature (Bio1), Mean Diurnal Range (Bio2), Max Temperature of Warmest Month (Bio5), Min Temperature of Coldest Month (Bio6), Annual Precipitation (Bio12), Precipitation of Wettest Quarter (Bio16), and Precipitation of Driest Quarter (Bio17) were the seven predictor variables modeled with up to 100% cumulative contributions for all models (Tables S2–S7). Bio1, Bio5, Bio12, Bio16 and Bio17 contributed most to *S. frugiperda* models, while parasitoid models top predictor variables were Bio1, Bio2, Bio6, Bio12, Bio16 globally (Tables S2–S7; Figures S1–S4).

### 3.2. Variable Importance

The jack-knife test of variable importance assesses which variable contributes most to the model output when used in isolation. From this study, annual mean temperature (Bio1) had the highest gain across all the models, which indicates that the variable contains useful information with regard to the species habitat suitability (Figures S1–S4).

### 3.3. FAW Habitat Suitability

**Figure 1:**
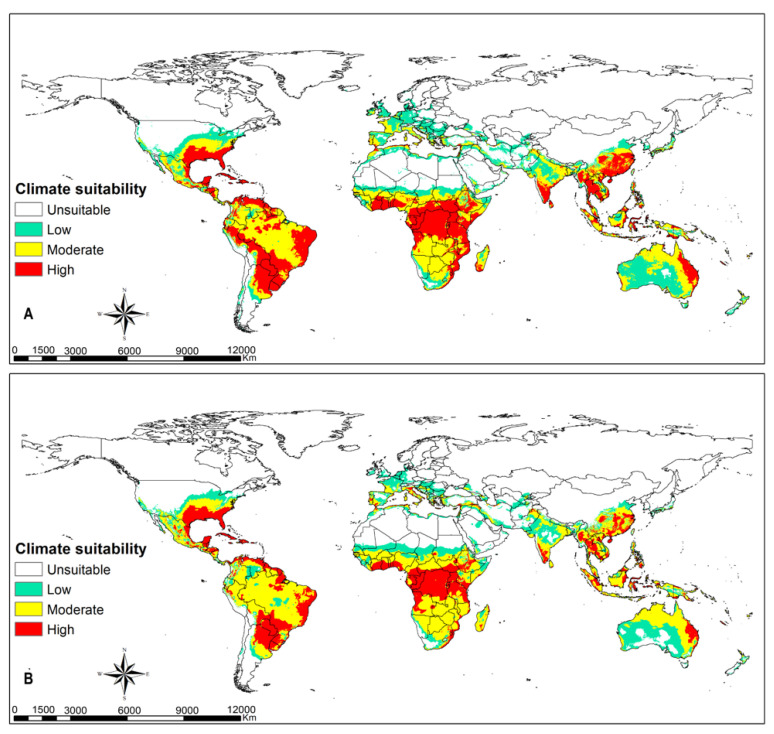
Potential bioclimatic suitability of *Spodoptera frugiperda* at a global extent using native and invaded population at (**A**) current climatic conditions and at (**B**) representative concentration pathway (RCP) 8.5 climatic scenario.

**Figure 2:**
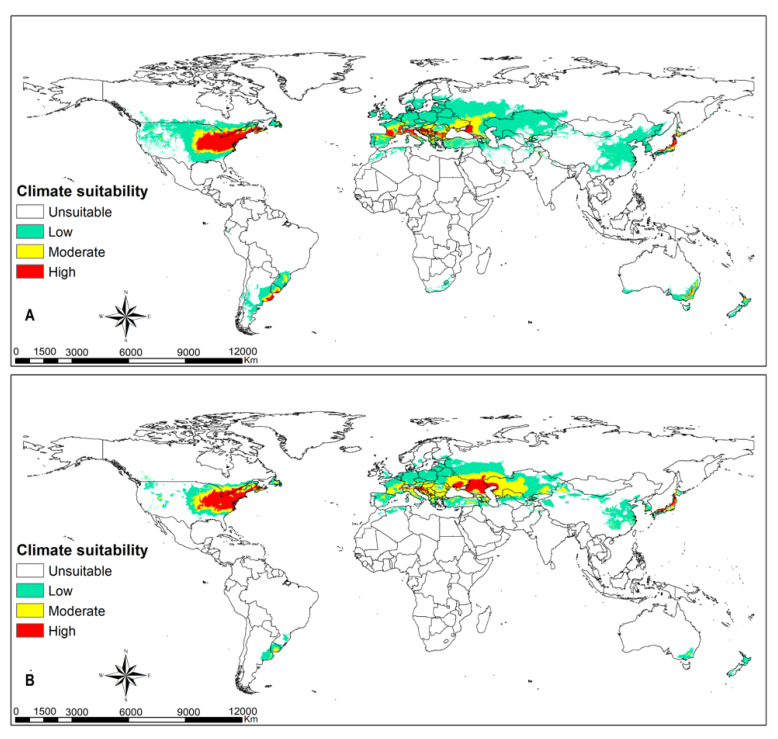
Potential areas suitable for *Spodoptera frugiperda*’s migration at a global extent using temporary migrated population at (**A**) current climatic conditions and at (**B**) RCP8.5 climatic scenario.

Greater parts of terrestrial ecosystems are predicted to be suitable habitats for FAW, except xeric environments in tropical and subtropical regions, and large parts of temperate and boreal ecosystems (Figure 1 and Figure 2). Highly suitable habitats comprise the coastal belt of West Africa from Côte d’Ivoire (Ivory Coast) to Nigeria; the Congo basin to eastern Africa; eastern, southern and southeastern Asia; some portions of eastern Australia; and western and southern Europe (Figure 1A). Most of these habitats will remain suitable despite climate change (RCPs 8.5), including FAW hotspots in the Americas (Figure 1B). The warmer parts of Europe can sustain the population of FAW, especially in the event of accidental pest introduction (Figure 1A). The results in Figure 1 confirm that there is high climate suitability for fall armyworm to survive in southeastern parts of United States of America, most parts of Latin America, sub-Saharan Africa, northern Australia, India, and southeast Asia. The intensity and extent of suitability vary across the different climatic scenarios. Figure 2 shows that regions in eastern parts of the United States of America, southern Europe and southernmost portions of western and eastern Europe, the Caucasian region and Japan have high climate suitability to host temporary migration of fall armyworm (Figure 2A). With climate change, the risk for hosting migratory populations might decline in Europe but increase in the Caucasian region and Japan (Figure 2B).

### 3.4. Parasitoids Habitat Suitability

**Figure 3:**
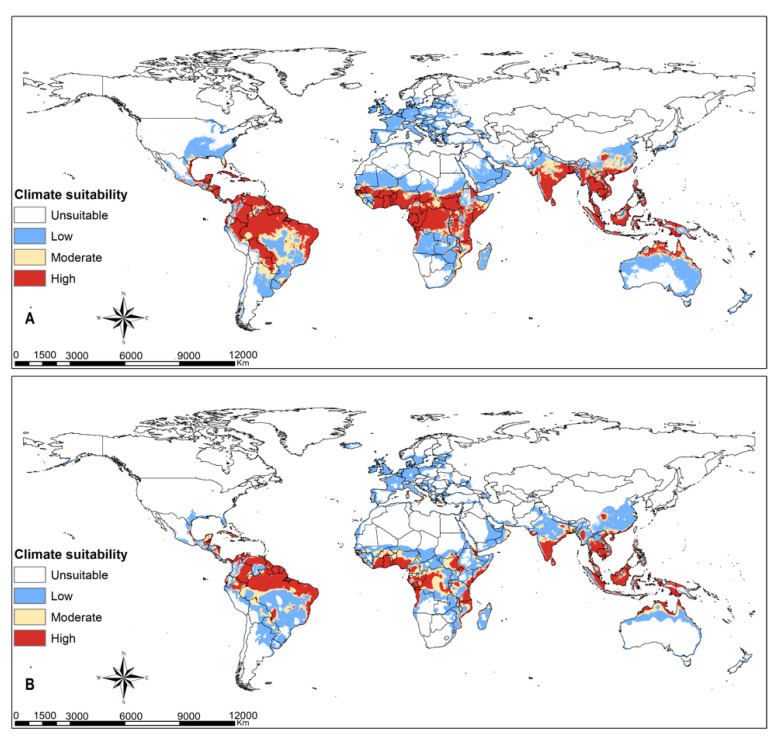
Predicted current and future habitat suitability for the egg parasitoid *Telenomus remus* at (**A**) current climatic conditions and at (**B**) RCP8.5 climatic scenario.

**Figure 4:**
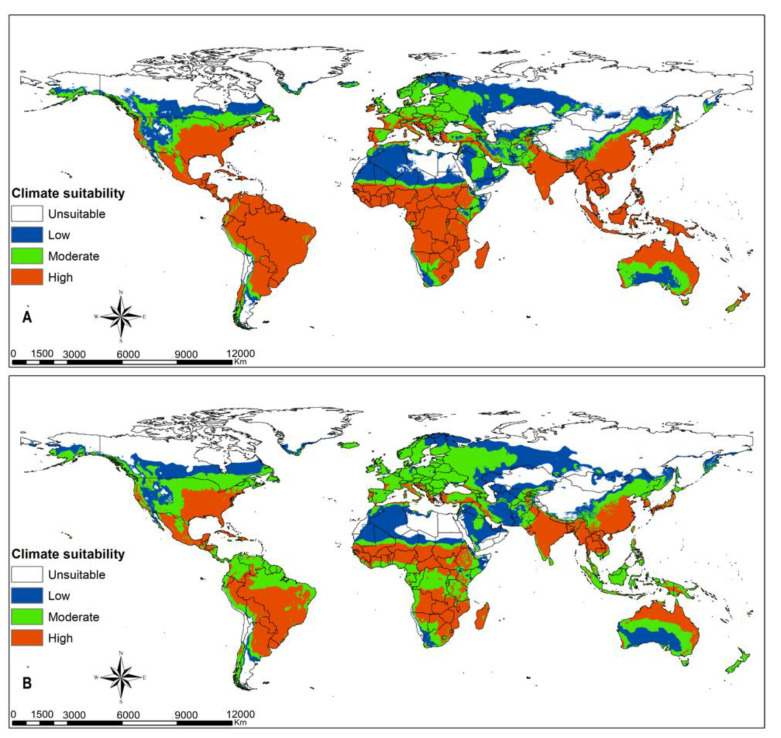
Predicted current and future habitat suitability for the egg parasitoid *Trichogramma pretiosum* at (**A**) current climatic conditions and at (**B**) RCP8.5 climatic scenario.

**Figure 5:**
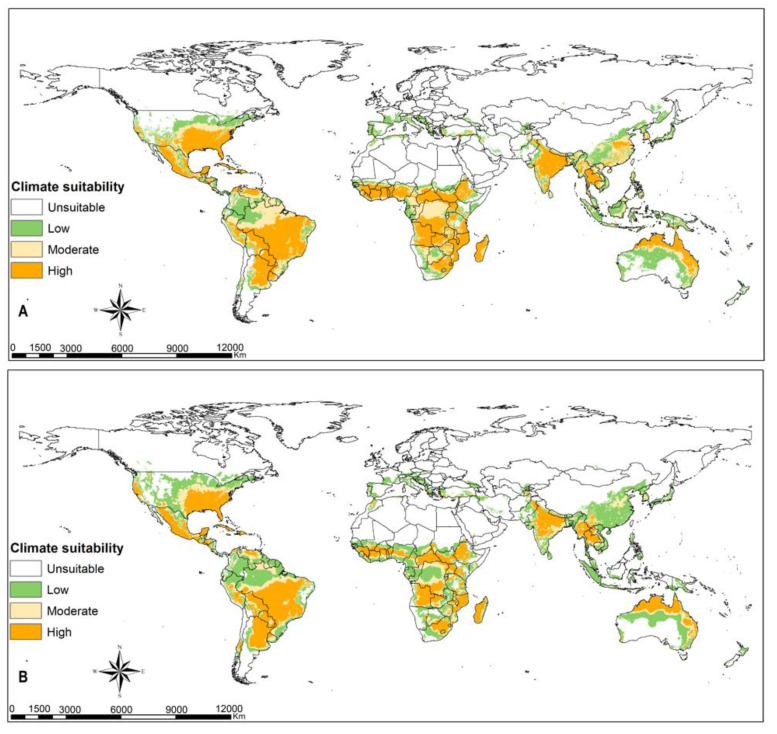
Predicted current and future habitat suitability for the egg-larval parasitoid *Chelonus insularis* at (**A**) current climatic conditions and at (**B**) RCP8.5 climatic scenario.

**Figure 6:**
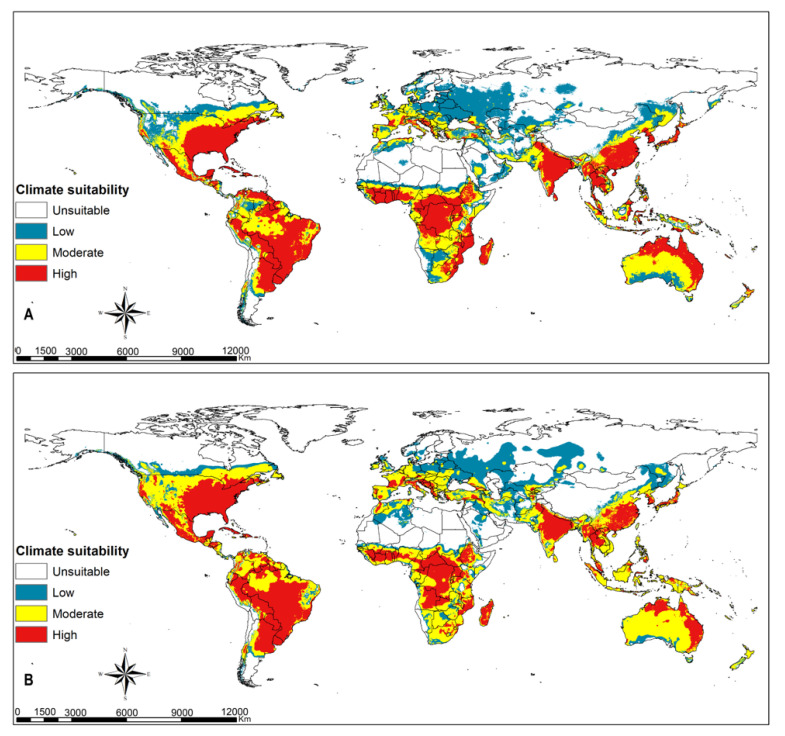
Predicted current and future habitat suitability for the larval parasitoid *Cotesia marginiventris* at (**A**) current climatic conditions and at (**B**) RCP8.5 climatic scenario.

**Figure 7:**
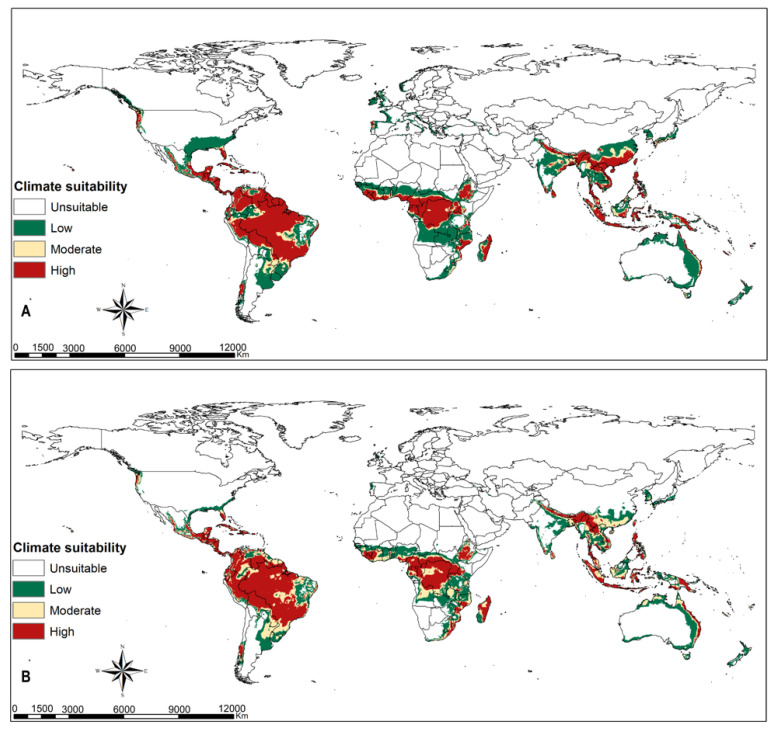
Predicted current and future habitat suitability for the larval parasitoid *Eiphosoma laphygmae* at (**A**) current climatic conditions and at (**B**) RCP8.5 climatic scenario.

**Figure 8:**
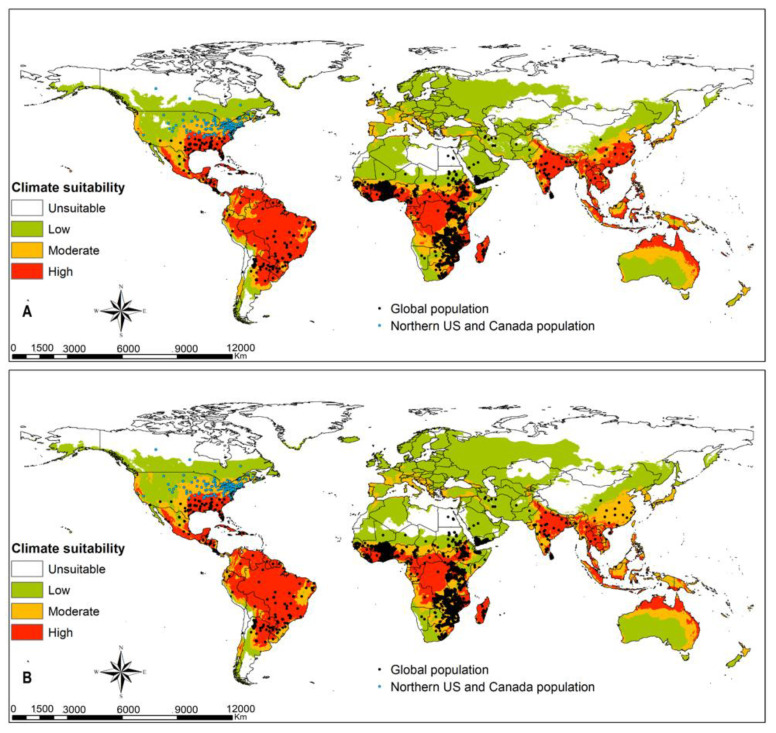
Georeferenced records for *Spodoptera frugiperda* combined with predicted habitat suitability for its parasitoids (all species combined); *Telenomus remus*, *Trichogramma pretiosum*, *Chelonus insularis*, *Cotesia marginiventris* and *Eiphosoma laphygmae* at (**A**) current climatic conditions and at (**B**) RCP8.5 climatic scenario.

**Figure 9:**
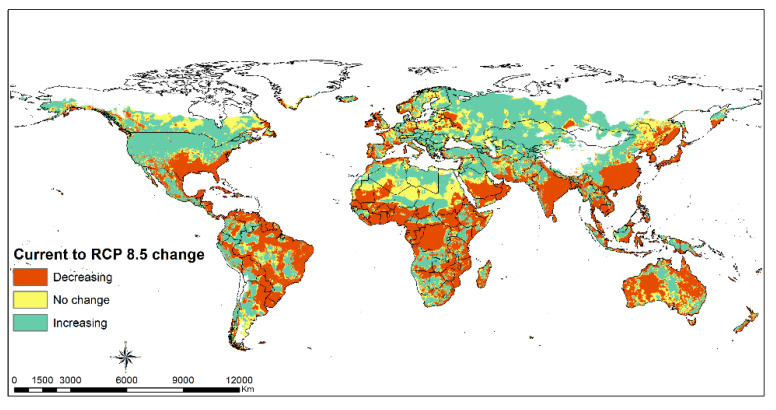
Habitat suitability range shift from current climate conditions to RCP8.5 climate scenario of an ensemble model of the five selected parasitoids; *Telenomus remus*, *Trichogramma pretiosum*, *Chelonus insularis*, *Cotesia marginiventris* and *Eiphosoma laphygmae*.

Tropical dry, moist and rainforest habitats are suitable for *Te. remus* (Figure 3A). Climate change will slightly decline the habitats suitable for the egg parasitoid *Te. remus* in the middle of Africa, south Asia and tropical South America (Figure 3B). *Trichogramma pretiosum* has a larger habitat suitability compared to *Te. remus*. Its potential distribution under current climate conditions (Figure 4A) overlaps suitable habitats for FAW, and this is anticipated to be the same under changing climate scenarios, RCP8.5 (Figure 1, Figure 2 and Figure 4A,B). Habitat suitability for *Ch. insularis* mirrors that of FAW except for the unsuitable habitats in Europe and insular regions of southeastern Asia (Figure 1, Figure 2 and Figure 5A). Eastern Asia may become less suitable for the egg-larval parasitoid with climate change, RCP8.5 (Figure 5A,B). The suitability maps for the larval parasitoid, *Co. marginiventris* are almost identical to that of the egg parasitoid *Tr. pretiosum* (Figure 4A,B and Figure 6A,B). A great portion of western and southern Europe is highly suitable for *Co. marginiventris* (Figure 6A). Together with *Tr. pretiosum*, *Co. marginiventris* is predicted to survive in harsh conditions of tropical shrubland (Figure 4A,B and Figure 6A,B). The habitat suitability of *Co. marginiventris* will not change despite global warming, RCP8.5 (Figure 6A,B). The best habitats for the larval parasitoid, *E. laphygmae* are restricted to the Amazon basin in South America, Central Africa and some coastal regions in west and southern Africa, eastern Asia and insular regions of South East Asia and Oceania (Figure 7A). This is likely to be stable with climate change (Figure 7B). Figure 8A,B are the maps of all combined parasitoids’ suitability areas overlaid with FAW’s maps. Most native and invasive regions suitable for FAW are also suitable for the selected parasitoids. However, most regions in the Europe, especially southern Europe, are only low to moderately suitable for the FAW parasitoids. The ensemble suitability for the selected parasitoids during the current climate conditions and with climate change, RCP8.5, is presented in Figure 8A,B. The figure shows that the northern region of Africa, north United States of America (USA), south Canada and large parts of Asia and Europe are less suitable for the five selected parasitoids, while the east and central Africa, South America are highly suitable. On the other hand, the results showed that the habitat suitability range shift for the five selected parasitoids will increase in the above-mentioned regions using the RCP8.5 climate change scenario (Figure 9).

## 4. Discussion

### 4.1. Model Evaluations

Despite the fact that our models demonstrated good results based on a bioclimate envelope approach, several previous assessments raised some limitations on the validity of this method [39]. Among factors determining species distributions and distribution change dynamics are biotic interactions (such as host plant or host/prey availability), evolutionary change and dispersal ability. These predictors are absent in the bioclimate envelope-based model approach. For instance, it is questionable to which extent future climates, together with anthropogenic landscape management, will influence the regulation of pests by natural enemies [40]. Another pitfall of the method is the integration in the model of factors such as irrigation. Significant effect of these factors might lead to a mismatch between host plants, pests and natural enemies in space and time, therefore decreasing the establishment likelihoods of biological control agents [41]. Nonetheless, it is agreed that the bioclimate envelope approach can provide useful first estimates and certainly guide decision making at the current time [38,42].

### 4.2. FAW Habitat Suitability

The FAW models developed are, in general, consistent with the CLIMEX output [9], and partly in agreement with the ensemble Species Distribution Model (SDM) [8] presented in previous assessments. FAW is already confirmed in Egypt [43] and more recently in Mauritania [44], Jordan and the United Arab Emirates [6]. A potential invasion route from there to Europe can be via the Middle East and Turkey. FAW moths can fly up to 50 km overnight. Thus, the migration of the pest over the 14 km width of the Gibraltar strait from the northernmost parts of Africa to reach southern Europe is also likely. Alongside the present work, previous pest risk assessments (PRAs) supported that climatic conditions in portions of southern Europe may be particularly suitable to FAW establishment as, for example, in Spain, Italy and Greece [10]. Subtropical dry forests in southern Europe and portions of temperate oceanic forests in western Europe are highly suitable habitats for FAW. The western and southern regions of the European continent and a few other temperate locations can, temporarily, be suitable habitats for FAW in the event of pest introductions and outbreaks.

In the southern Hemisphere, our predictions indicate the suitability for the establishment of FAW in Australia, especially in Queensland and in some Oceania island countries. This is in line with the recent reports of FAW in Australia [45] and Timor Leste [46] in 2020. Our models categorize the North Island of New Zealand as of low suitability to FAW under current the climate, but report that the region may become moderately suitable in the event of RCP8.5 climate conditions.

The models developed confirm to a large extent the suitability of tropical moist forests and rainforests and subtropical humid forests for FAW establishment. Tropical and subtropical dry forests are also suitable habitats to FAW. However, the pest does not diapause and has a minimum temperature threshold of 12.6 °C for egg-to-adult development [47]. Therefore, FAW will not survive winter conditions in most of Europe, and further outbreaks can only originate from the closest regions with permanent multiple-generation habitat suitability status. All the regions mentioned above will generally remain suitable with a 3.7 °C temperature increase (RCP8.5 climate change scenario). Under this scenario, probable moderate habitat suitability expansion to eastern outermost parts of tropical shrubland in Australia is expected. Western Europe may potentially become less suitable under RCP8.5 climatic conditions. The North Island of New Zealand may become moderately suitable in the event of RCP8.5 climates.

### 4.3. Parasitoid Habitat Suitability

*Trichogramma pretiosum* will survive increased temperatures of RCP8.5 and is, therefore, predicted to be the most resilient egg parasitoid species compared to *Te. remus*. However, the searching capabilities and oviposition rates of *Tr. pretiosum* are estimated to be less than that of *Te. remus* [12,48,49,50]. Hence further assessment, including biological parameters of these two egg parasitoids, using tools such as Insect Life Cycle Modeling (ILCYM) or CLIMEX can highlight their potential for FAW management. Our models suggest that if empirically proven efficient, *Tr. pretiosum* (egg parasitoid) and *Co. marginiventris* (larval parasitoid) can be deployed together sequentially to enhance the success of biocontrol programs in most of the areas affected by FAW. Similar sequential deployment for *Te. remus* and *E. laphygmae* could be beneficial. The egg-larval parasitoid *Ch. insularis,* and to a lesser extent *E. laphygmae, Te. remus* and *Ch. insularis,* may be considered as additional candidate parasitoid in such programs. *Trichogramma pretiosum* and *Co. marginiventris* can be candidate biocontrol agents for vast regions of the Sahel, particularly northern Guinea and semiarid/Sudan savannas. Interestingly, tropical and subtropical regions of Australia are also highly suitable for *Co. marginiventris*. Parts of the temperate regions of the country can also be suitable for the parasitoid, particularly under climate change. The North Island of New Zealand is potentially suitable for the establishment of *Co. marginiventris* independent of climatic conditions (current, and RCP8.5), although the risks of FAW to invade this region are predicted to be low for the current and future climate. Combined maps of all parasitoids support that the selected parasitoids might be good candidates for biocontrol programs of FAW globally.

Establishment of hymenopteran parasitoids depends on a number of factors. For instance, it is reported that dispersal patterns of *Te. remus* are highly influenced not only by wind and vegetation structure, but also by temperature and humidity [21]. Successful dispersal of *Trichogramma* species also depends on multiple factors, including weather conditions, host, intraguild predation and pesticide use. Our models generally support the potential of the five parasitoids to establish in critical FAW hotspots, but we do recognize that the limited availability of the parasitoid species’ records (Table S1) might constrain the accuracy of the climate modeling experiment presented in this effort. However, inundative releases of egg parasitoids in habitats with FAW risks can enhance the effectiveness of long-term management of FAW.

### 4.4. Parasitoids Potential for Biocontrol

As a new invasive pest species, FAW has been introduced into Africa without the rich guild of parasitoids naturally occurring in its native range in the tropical and subtropical Americas [14]. One exception to this is the parasitoid *Te. remus* that has been recovered in several countries in western, eastern and southern Africa, where it is now regularly found on FAW egg masses [29,30,51]. The species, described initially from Malaysia, was introduced in the 70s into the Caribbean against *Spodoptera* spp. [52] and is widespread in the Americas, with records in 14 countries [14]. Most recent records indicate its presence on FAW eggs in the Guangdong province in southern China [30] where FAW has recently spread. The first data of field parasitism by *Te. remus* presently measured in Benin and Ghana [30] show 14.5–25.9% attack on egg masses lower than those assessed in eastern Africa, which average above 50% [51]. This suggests a better performance of *Te. remus* at lower temperatures due to a longer duration of the egg stage, and thereby a larger window of opportunity for parasitism. The possibility to also use the two egg parasitoids *Te. remus* and *Tr. pretiosum* in augmentative releases, as successfully practiced in South America [20,53], makes them less dependent on the predicted distribution. This is true in situations where local mass production allows routine field releases in areas where climatic conditions are only suitable part of the year but which can steadily be exploited by FAW due to its high migratory capacity.

Conversely, the present prediction models are particularly relevant for the solitary larval and egg-larval parasitoids, *Co. marginiventris*, *Ch. insularis* and *E. laphygmae* that cannot be produced in comparatively large numbers, as mass rearing of FAW caterpillars is particularly challenging because of the cannibalistic behavior of the larvae [54]. The solitary larval parasitoid, *E. laphygmae* is a valuable addition among the parasitoid candidates as the species is rather host-specific [55] and its action on older larvae extends the window time of parasitism during FAW larval development. The models, however, predict a relatively narrow climatic niche for *E. laphygmae* compared to other parasitoids considered here. The inoculative release of *Co. marginiventris*, *Ch. insularis* and *E. laphygmae* may well complement the action of conspecific parasitoids native to Africa, such as *Cotesia icipe* Fernández-Triana & Fiaboe, *Chelonus bifoveolatus* Szépligeti and *Chelonus curvimaculatus* Cameron, showing disparate distributions and/or various control performance on FAW [30,51] (though, the present work did not provide insights on the performance of these parasitoids to control FAW). In addition, possible interspecific competition between native and exotic species, as well as the role of alternative lepidopteran hosts of these parasitoids in Africa, deserve further attention. Therefore, efforts to study the behavioral and biological characteristics of each of the respective native species have been initiated to better assess specific parasitoid niches and to provide the decision basis for planning introductions in new geographic areas to maximize the impact against FAW.

## 5. Conclusions

Tropical and subtropical regions of both Asia, Africa and Australia are highly suitable for FAW establishment under current and future climate scenarios. Subtropical dry forests in southern Europe and portions of temperate oceanic forests in western Europe are also highly suitable habitats for FAW. Our models categorize the North Island of New Zealand and temperate regions of Europe and Asia as of low suitability for FAW under current and future climates. However, some of these regions may become suitable in the event of RCP8.5 climate conditions. However, under the current climatic scenario, FAW outbreaks in these unsuitable habitats, from its migration from close regions with permanent multiple-generation habitat suitability status, is a possibility that needs to be monitored regularly. All the five hymenopteran parasitoids considered show particularly high establishment potential in most of the FAW-affected areas and those of potential invasion risks. These habitats can potentially be important sites for biocontrol implementation. However, the establishment will also depend on the role of alternative host plants in the absence of maize, along with food production systems and dispersal and survival capabilities of the biocontrol agents. Moreover, a considerable amount of work is required to ensure that the introduction of a parasitoid as a classical biological control agent would not result in unintended and damaging effects on nontarget species. Our models provide the first estimates to guide decision making for biological control-based medium and long-term management of FAW globally. However, it is still questionable how host-enemy synchrony will be sustained in the face of changing temperatures, and how this will determine FAW outbreaks in critical regions.

## Figures and Tables

**Table 1:** Outline of the two CMIP5′ global cimate models (GCMs) tested.

GCM	Institution	Horizontal Resolution	2× [CO_2_] Equilibrium Climate Sensitivity (°C)
GISS-E2-R *	National Aeronautics and Space Association Goddard Institute for Space Studies (NASA GISS)	2° × 2.5°	2.1
HadGEM2-ES *	UK Meteorological Office—Hadley Centre	1.25° × 1.875°	4.6

* HadGEM2-ES (4.6 °C climate sensitivity) is among the warmer CMIP5 models for almost all locations, while GISS-E2-R (2.1 °C) inclines to be relatively cool over much of the land area [36].

**Table 2:** Area under the curve (AUC) values of Maxent’s fall armyworm (FAW) models.

*Spodoptera frugiperda*	Current		RCP8.5	
	Training	Test	Training	Test
Native and invaded regions model	0.929	0.925	0.902	0.913
Temporary migrated regions model	0.952	0.942	0.951	0.931

**Table 3:** AUC values of Maxent’s parasitoid models.

Parasitoid Species	Current		RCP8.5	
	Training	Test	Training	Test
*Chelonus insularis*	0.934	0.953	0.920	0.941
*Cotesia marginiventris*	0.900	0.946	0.869	0.958
*Eiphosoma laphygmae*	0.969	0.971	0.965	0.966
*Telenomus remus*	0.931	0.956	0.916	0.917
*Trichogramma pretiosum*	0.856	0.864	0.802	0.807

## Supplementary Materials

The following are available online at https://www.mdpi.com/2075-4450/12/4/273/s1. Figure S1: Jackknife estimates for *Spodoptera frugiperda* (a) for the native and invaded population model (A) Current conditions (B) RCP8.5 and (b) for the temporary migrated population model (A) Current conditions (B) RCP8.5. Figure S2: Jackknife estimates for the egg parasitoids *Telenomus remus* and *Trichogramma pretiosum* under current and future RCP8.5 climates. Figure S3: Jackknife estimates for the egg-larval parasitoid *Chelonus insularis* under current and future RCP8.5 climates. Figure S4: Jackknife estimates for the larval parasitoids *Cotesia marginiventris* and *Eiphosoma laphygmae* under current and future RCP8.5 climates. Table S1: Number of presence records of FAW’s parasitoids used in the models. Table S2: Percentage contribution of environmental predictor variables used for FAW *Spodoptera frugiperda* models. Table S3: Percentage contribution of environmental predictor variables used for the egg-parasitoid *Telenomus remus* models. Table S4: Percentage contribution of environmental predictor variables used for the egg-parasitoid *Trichogramma pretiosum* models. Table S5: Percentage contribution of environmental predictor variables used for the egg-larval parasitoid *Chelonus insularis* models. Table S6: Percentage contribution of environmental predictor variables used for the larval parasitoid *Cotesia marginiventris* models. Table S7: Percentage contribution of environmental predictor variables used for the larval parasitoid *Eiphosoma laphygmae* models. File S1: Presence records used for *Spodoptera frugiperda*. File S2: Presence records used for *Telenomus remus*. File S3: Presence records used for *Trichogramma pretiosum*. File S4: Presence records used for *Chelonus insularis*. File S5: Presence records used for *Cotesia marginiventris*. File S6: Presence records used for *Eiphosoma laphygmae*.
