# Supplementary material for: Global Habitat Suitability of Spodoptera frugiperda (JE Smith) (Lepidoptera, Noctuidae): Key Parasitoids Considered for Its Biological Control
Source: Insects. 2021 Mar 24;12(4):273. doi: 10.3390/insects12040273 (PMC8063841; doi:10.3390/insects12040273)
Supplement: Supplementary file 1 [file insects-12-00273-s001.zip › Supplementary materials/Tables S1-7/Table S2.pdf]

Table S2. Percentage contribution of environmental predictor variables used for *Spodoptera frugiperda* models

Code	Bioclimatic variables*	Unit	% contribution of environmental variables		
			Current	RCP8.5	
				GISS-E2-R	HadGEM2-ES
**Bio1**	**Annual Mean Temperature**	℃	17.7	2.6	18.0
**Bio2**	**Mean Diurnal Range (Mean of monthly (max temp - min temp))**	℃	0.9	1.1	0.3
**Bio5**	**Max Temperature of Warmest Month**	℃	27.2	18.3	1.7
**Bio6**	**Min Temperature of Coldest Month**	℃	1.0	0.5	0.7
**Bio12**	**Annual Precipitation**	mm	22.4	7.5	9.7
**Bio16**	**Precipitation of Wettest Quarter**	mm	15.7	1.4	19.4
**Bio17**	**Precipitation of Driest Quarter**	mm	15.1	12.8	6.0
**Total percentage contribution**			**100**	**100**	

* Bioclimatic variables in bold represent the intuitively preselected environmental layers that contributed best in final models.
