# Supplementary material for: Global Habitat Suitability of Spodoptera frugiperda (JE Smith) (Lepidoptera, Noctuidae): Key Parasitoids Considered for Its Biological Control
Source: Insects. 2021 Mar 24;12(4):273. doi: 10.3390/insects12040273 (PMC8063841; doi:10.3390/insects12040273)
Supplement: Supplementary file 1 [file insects-12-00273-s001.zip › Supplementary materials/Tables S1-7/Table S1.pdf]

S1 Table. Number of presence records of FAW's parasitoids used in the models

Species	Number of occurrences sourced from published data	Total number of presence points used in the models	References* (listed below)
Telenomus remus	45	66	[1-5]
Trichogramma pretiosum	1	59	[6]
Chelonus insularis	77	98	[5-19]
Cotesia marginiventris	12	60	[5,11,13,17,19-20]
Eiphosoma laphygmae	53	53	[11,18,21-30]

***References**

1. Agboyi LK, Goergen G, Beseh P, Mensah SA, Clottey VA, Glikpo R, et al. 2020. Parasitoid complex of fall armyworm, *Spodoptera frugiperda*, in Ghana and Benin. Insects 2020;11(2): 68. doi: 10.3390/insects11020068.

2. Kenis M, du Plessis H, Van den Berg J, Ba MN, Goergen G, Kwadjo KE, et al. *Telenomus remus*, a Candidate Parasitoid for the Biological Control of *Spodoptera frugiperda* in Africa, is already Present on the Continent. *Insects.* 2019;*10(4)*: 92. doi: 10.3390/insects10040092.

3. Liao YL, Yang B, Xu MF, Lin W, Wang DS, Chen KW, et al. First report of *Telenomus remus* parasitizing *Spodoptera frugiperda* and its field parasitism in southern China. J Hymenopt Res. 2019;73: 95-102.

4. HOL: various contributors. Hymenoptera Online (HOL); 2020 [Cited 2020 February 03]. Available from: https://hol.osu.edu.

5. Maes JM. Catálogo de los insectos controladores biológicos en Nicaragua. Vol. III. Insectos Parasitoides. Rev Nicar Entomol. 1989;10: 1-138.

6. Gutiérrez-Ramírez A, Robles-Bermúdez A, Cambero-Campos J, Santillán-Ortega C, Ortíz-Catón M, Coronado-Blanco JM, et al. Parasitoides de *Spodoptera frugiperda* (Lepidoptera: Noctuidae) Encontrados en Nayarit, México. Southwest Entomol. 1 September 2015;40(3): 555-564. doi: 10.3958/059.040.0314.

7. Bortolotto OC, Menezes Jr ADO, Hoshino AT, Carvalho MG, Pomari-Fernandes A, Salgado-Neto G. Sugar solution treatment to attract natural enemies and its impact on fall armyworm *Spodoptera frugiperda* in maize fields. Interciencia. 2014;39(6): 416-421.

8. Bridwell JC. List of Kansas Hymenoptera. Trans Kans Acad Sci. 1903;16: 203-211.

9. Canas LA, O'Neil RJ. Applications of sugar solutions to maize, and the impact of natural enemies on fall armyworm. Int J Pest Manage. 1998;44(2): 59-64.

10. Ehler LE. Impact of native predators and parasites on *Spodoptera exigua*, an introduced pest of alfalfa hay in northern California. Biocontrol. 2007;52(3): 323-338.

11. Jourdie V, Alvarez N, Turlings TCJ. Identification of seven species of hymenopteran parasitoids of *Spodoptera frugiperda*, using polymerase chain reaction amplification and restriction enzyme digestion. Agr Forest Entomol. 2008;10(2): 129-136.

12. Jourdie V, Virla E, Murillo H, Bento JMS, Turlings TC, Alvarez N. Phylogeography of *Chelonus insularis* (Hymenoptera: Braconidae) and *Campoletis sonorensis* (Hymenoptera: Ichneumonidae), two primary neotropical parasitoids of the fall armyworm (Lepidoptera: Noctuidae). Ann Entomol Soc Am. 2010;103(5): 742-749.

13. Marenco RA, Saunders JL. Parasitoides del gusano cogollero, *Spodoptera frugiperda* (Lepidoptera: Noctuidade) en maíz en Turrialba, Costa Rica. Manejo Integrado Plagas. 1993;27: 18-23.

14. Molina-Ochoa J, Carpenter JE, Lezama-Gutiérrez R, Foster JE, González-Ramírez M, Angel-Sahagún CA et al. Natural distribution of hymenopteran parasitoids of *Spodoptera frugiperda* (Lepidoptera: Noctuidae) larvae in Mexico. Fla Entomol. 2004;87(4): 461-472.

15. Murúa G, Molina-Ochoa J, Coviella C. Population dynamics of the fall armyworm, *Spodoptera frugiperda* (Lepidoptera: Noctuidae) and its parasitoids in northwestern Argentina. Fla Entomol. 2006;89(2): 175-182.

16. Murúa MG, Molina-Ochoa J, Fidalgo P. Natural distribution of parasitoids of larvae of the fall armyworm, *Spodoptera frugiperda*, in Argentina. J Insect Sci. 2009;9(1): 20.

17. Riggin TM, Wiseman BR, Isenhour DJ, Espelie KE. Incidence of Fall Armyworm (Lepidoptera: Noctuidae) Parasitoids on Resistant and Susceptible Com Genotypes. Environ Entomol. 1992;21(4): 888-895.

18. Wheeler GS, Ashley TR, Andrews KL. Larval parasitoids and pathogens of the fall armyworm in Honduran maize. Entomophaga. 1989;34: 331-340.

19. Wilson JW. The biology of parasites and predators of *Laphygma exigua* Huebner reared during the season of 1932. Fla Entomol. 1933;17: 1-15.

20. Molina-Ochoa J, Carpenter JE, Heinrichs EA, Foster JE. Parasitoids and parasites of *Spodoptera frugiperda* (Lepidoptera: Noctuidae) in the Americas and Caribbean Basin: an inventory. Fla Entomol. 2003;86(3): 254-289.

21. Fernandez-Triana JL, Grillo-Ravelo H. A taxonomic review of Cuban *Eiphosoma* Cresson (Hymenoptera: Ichneumonidae), with biogeographical notes. Zootaxa. 2007;1655: 49-61.

22. Gauld ID. The Ichneumonidae of Costa Rica, 3. Mem Amer Ent Inst. 2000;63: 1-453.

23. González-Moreno A, Bordera S. The Ichneumonidae (Hymenoptera: Ichneumonoidea) of Ría Lagartos Biosphere Reserve, Yucatán, Mexico. Zootaxa. 2012;3230(1): 1. doi:10.11646/zootaxa.3230.1.1.

24. Melo IF. Espécies de *Eiphosoma* Cresson, 1865 (Hymenoptera, Ichneumonidae, Cremastinae) em áreas de mata e cerrado da bacia hidrográfica do rio Mogi-Guaçu. M.Sc. Thesis, Universidade Federal de São Carlos. 2011. Available from: https://repositorio.ufscar.br/handle/ufscar/2021.

25. Melo IF, Penteado-Dias AM. Estudo qualitativo das espécies de *Eiphosoma* Cresson, 1865 (Hymenoptera, Ichneumonidae, Cremastinae) em ecossistemas brasileiros. VI Congresso Internacional de Meio Ambiente da AUGM. São Carlos, SP: Anais de Eventos da UFSCar. 2009;5: 15.

26. Onody HC, de Melo IF, Penteado-Dias AM. Abundance, richness and diversity of *Eiphosoma* Cresson 1865 (Hymenoptera, Ichneumonidae) species associated with organic crops. Idesia. 2012;30(1): 115-120.

27. Shimbori EM, Onody HC, Fernandes DRR, Silvestre R, Tavares MT, Penteado-Dias AM. Hymenoptera “Parasitica” in the state of Mato Grosso do Sul, Brazil. Iheringia Sér Zool. 2017;107: 1-12.

28. Silveira JCF, Sasaki ET, Forner MA, Honda MS, Calafiori EMH. Ocorrencia de parasitoides de *Spodoptera frugiperda* (J.E. Smith, 1797) em cultura de milho, em Espirito Santo do Pinhal, SP. Ecossistema. 1987 ;12: 41-44.

29. Townes H, Townes M. A catalogue and reclassification of Neotropic Ichneumonidae. Mem Amer Ent Inst. 1966;8: 1-367.

30. Valicente FH. Levantamento dos inimigos naturais de *Spodoptera frugiperda* (J.E. Smith, 1797) (Lepidoptera: Noctuidae) em diferentes regioes de Minas Gerais. An Soc Entomol Brasil. 1989;18: 119-130.
