# Supplementary material for: Global Habitat Suitability of Spodoptera frugiperda (JE Smith) (Lepidoptera, Noctuidae): Key Parasitoids Considered for Its Biological Control
Source: Insects. 2021 Mar 24;12(4):273. doi: 10.3390/insects12040273 (PMC8063841; doi:10.3390/insects12040273)
Supplement: Supplementary file 1 [file insects-12-00273-s001.zip › Supplementary materials/Tables S1-7/Table S7.pdf]

**Table S7. Percentage contribution of environmental predictor variables used for the larval parasitoid *Eiphosoma laphygmae* models**

| Code | Bioclimatic variables* | Unit | % contribution of environmental variables | | |
|---|---|---|---|---|---|
| | | | Current | RCP8.5 | |
| | | | | GISS-E2-R | HadGEM2-ES |
| **Bio1** | **Annual Mean Temperature** | ℃ | 0.9 | 0.5 | 1.7 |
| **Bio2** | **Mean Diurnal Range (Mean of monthly (max temp - min temp))** | ℃ | 3 | 0 | 8.6 |
| **Bio5** | **Max Temperature of Warmest Month** | ℃ | 0.1 | 0 | 1.7 |
| **Bio6** | **Min Temperature of Coldest Month** | ℃ | 5.4 | 8.4 | 0.6 |
| **Bio12** | **Annual Precipitation** | mm | 18.2 | 8.6 | 4.9 |
| **Bio16** | **Precipitation of Wettest Quarter** | mm | 62.8 | 0 | 56.3 |
| **Bio17** | **Precipitation of Driest Quarter** | mm | 9.6 | 8.7 | 0 |
| **Total percentage contribution** | | | **100** | **100** | |

* Bioclimatic variables in bold represent the intuitively preselected environmental layers that contributed best in final models
