# Supplementary material for: Global Habitat Suitability of Spodoptera frugiperda (JE Smith) (Lepidoptera, Noctuidae): Key Parasitoids Considered for Its Biological Control
Source: Insects. 2021 Mar 24;12(4):273. doi: 10.3390/insects12040273 (PMC8063841; doi:10.3390/insects12040273)
Supplement: Supplementary file 1 [file insects-12-00273-s001.zip › Supplementary materials/Tables S1-7/Table S6.pdf]

Table S6. Percentage contribution of environmental predictor variables used for the larval parasitoid *Cotesia marginiventris* models

Code	Bioclimatic variables*	Unit	% contribution of environmental variables		
			Current	RCP8.5	
				GISS-E2-R	HadGEM2-ES
**Bio1**	**Annual Mean Temperature**	℃	0.3	0	27.6
**Bio2**	**Mean Diurnal Range (Mean of monthly (max temp - min temp))**	℃	0.4	0.1	10.1
**Bio5**	**Max Temperature of Warmest Month**	℃	27.7	0.1	9.6
**Bio6**	**Min Temperature of Coldest Month**	℃	9.2	4.4	0
**Bio12**	**Annual Precipitation**	mm	59.4	44.7	0
**Bio16**	**Precipitation of Wettest Quarter**	mm	0	0	0
**Bio17**	**Precipitation of Driest Quarter**	mm	3	0.8	2.6
**Total percentage contribution**			**100**	**100**	

* Bioclimatic variables in bold represent the intuitively preselected environmental layers that contributed best in final models.
