# Supplementary figures and images for: Global Habitat Suitability of Spodoptera frugiperda (JE Smith) (Lepidoptera, Noctuidae): Key Parasitoids Considered for Its Biological Control
Source: Insects. 2021 Mar 24;12(4):273. doi: 10.3390/insects12040273 (PMC8063841; doi:10.3390/insects12040273)

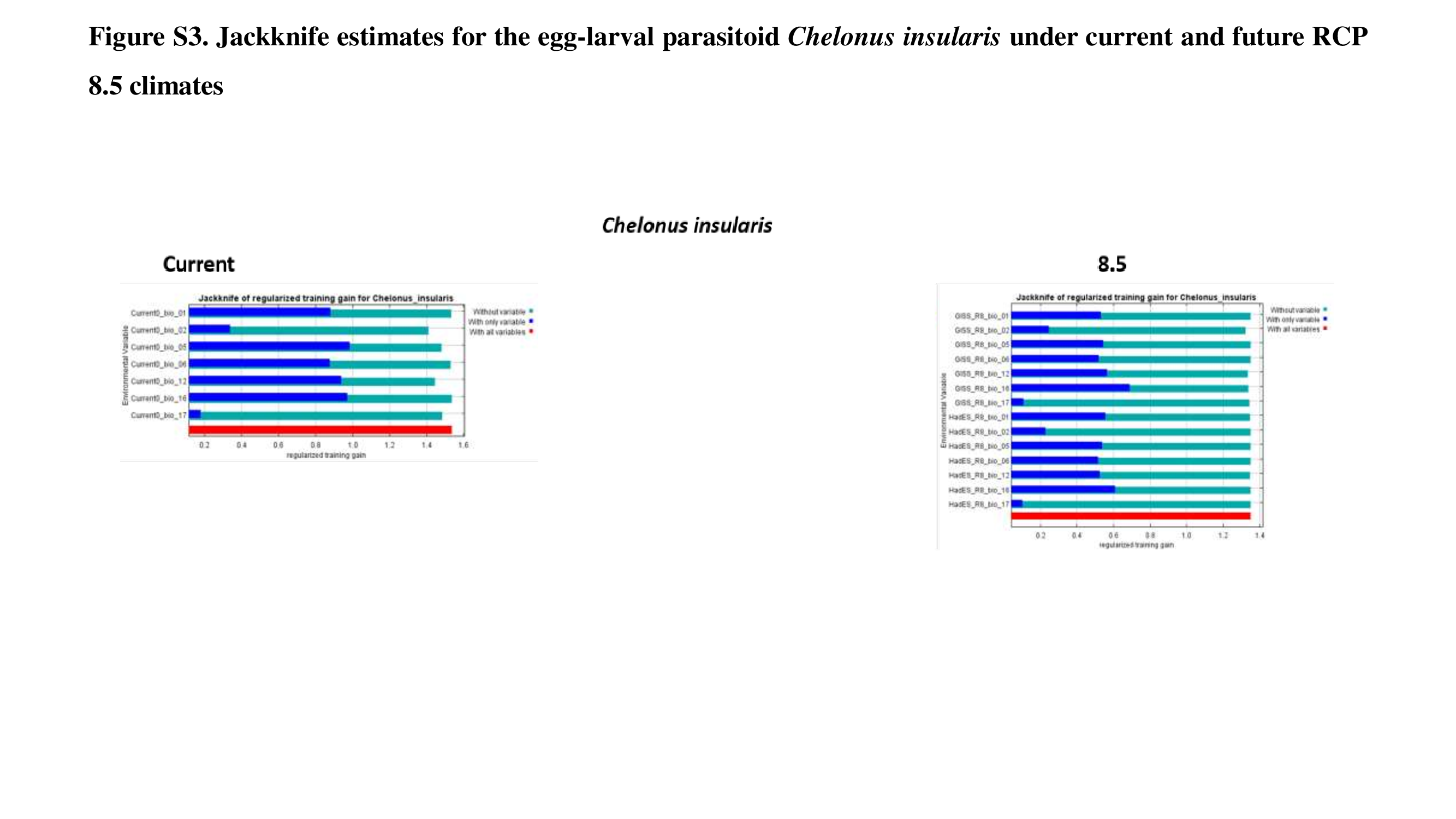

Supplement: Supplementary file 1 [file insects-12-00273-s001.zip › Supplementary materials/Figures S1-4/Figure S3.tiff]

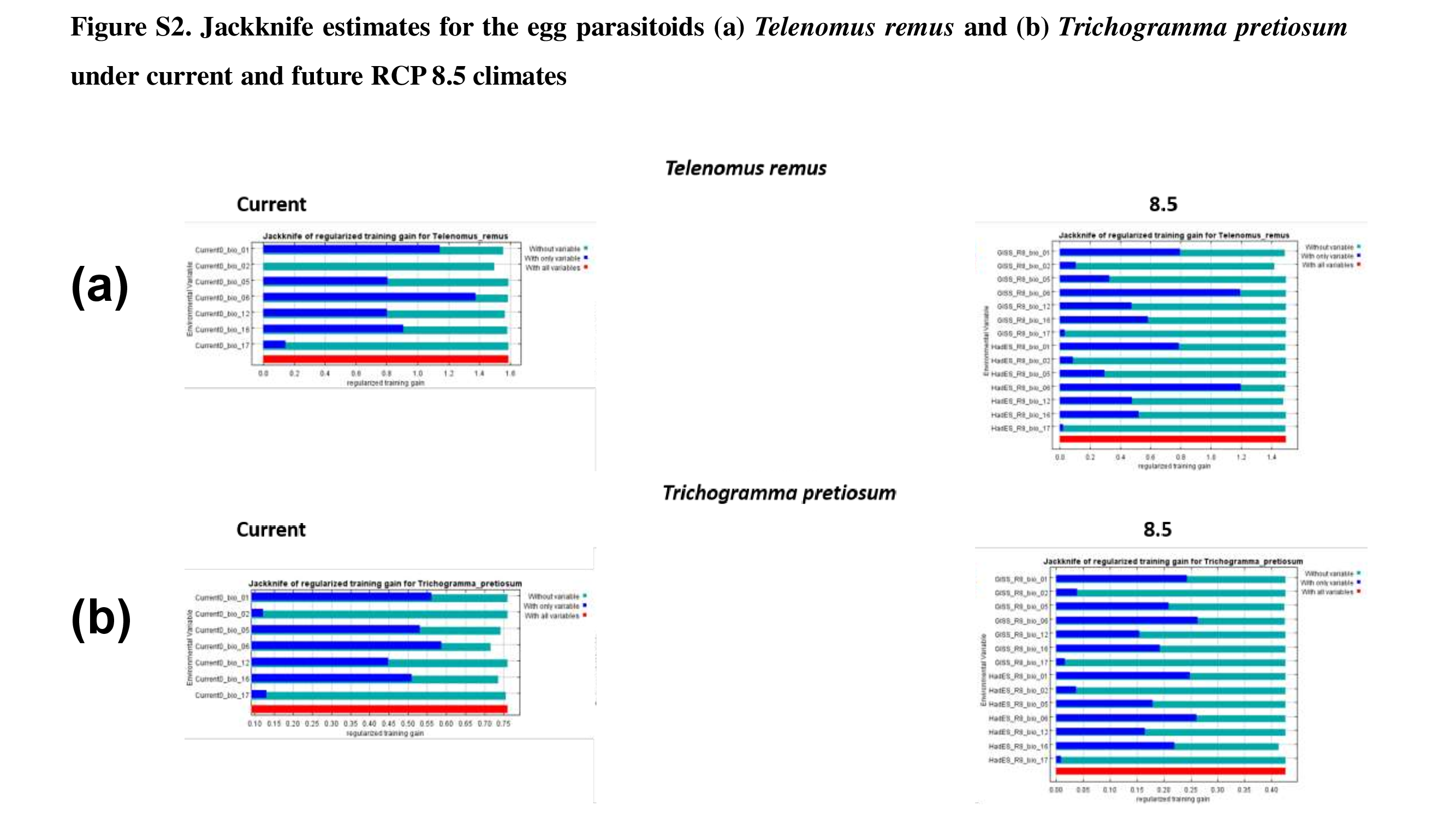

Supplement: Supplementary file 1 [file insects-12-00273-s001.zip › Supplementary materials/Figures S1-4/Figure S2.tiff]

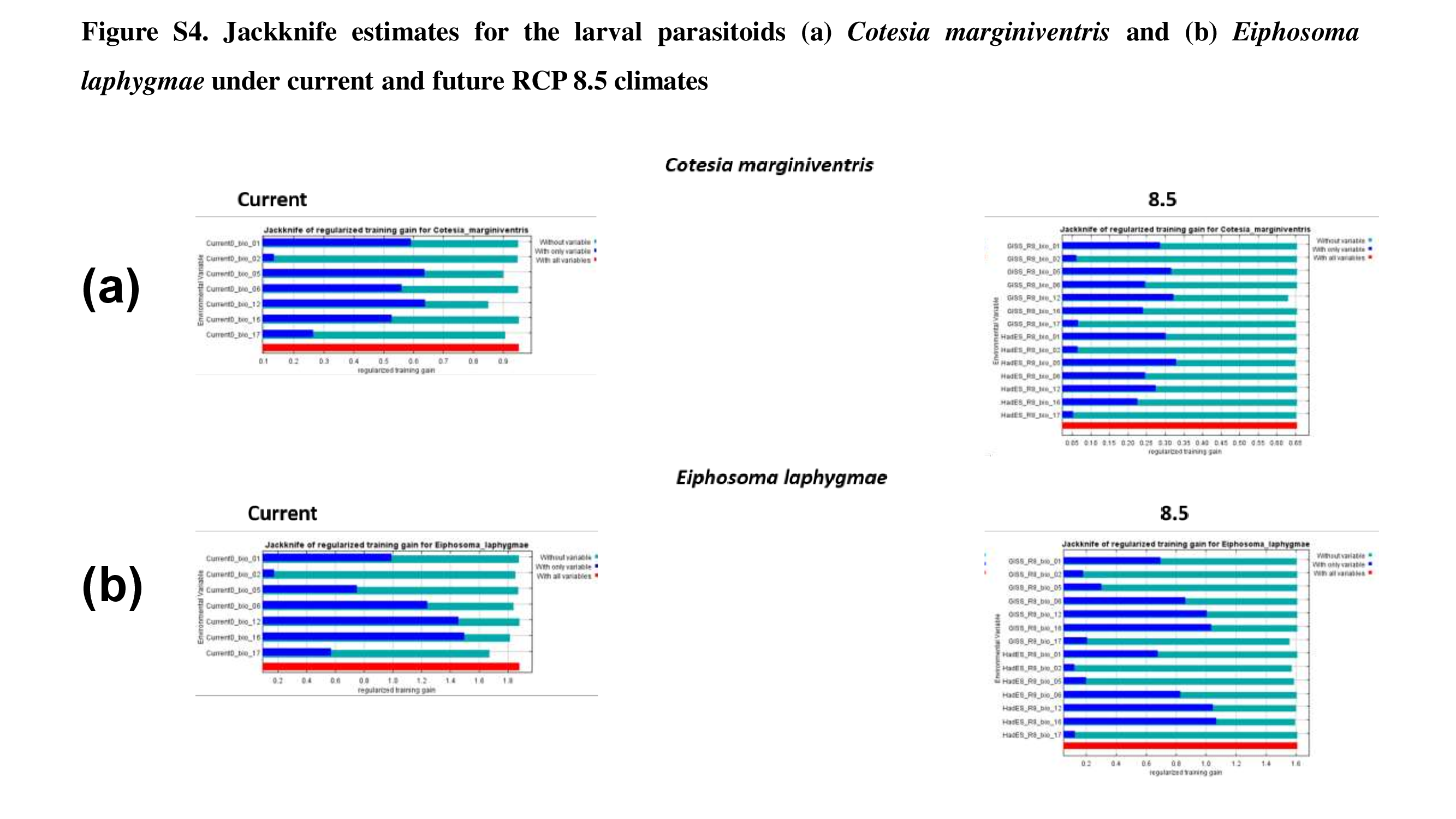

Supplement: Supplementary file 1 [file insects-12-00273-s001.zip › Supplementary materials/Figures S1-4/Figure S4.tiff]

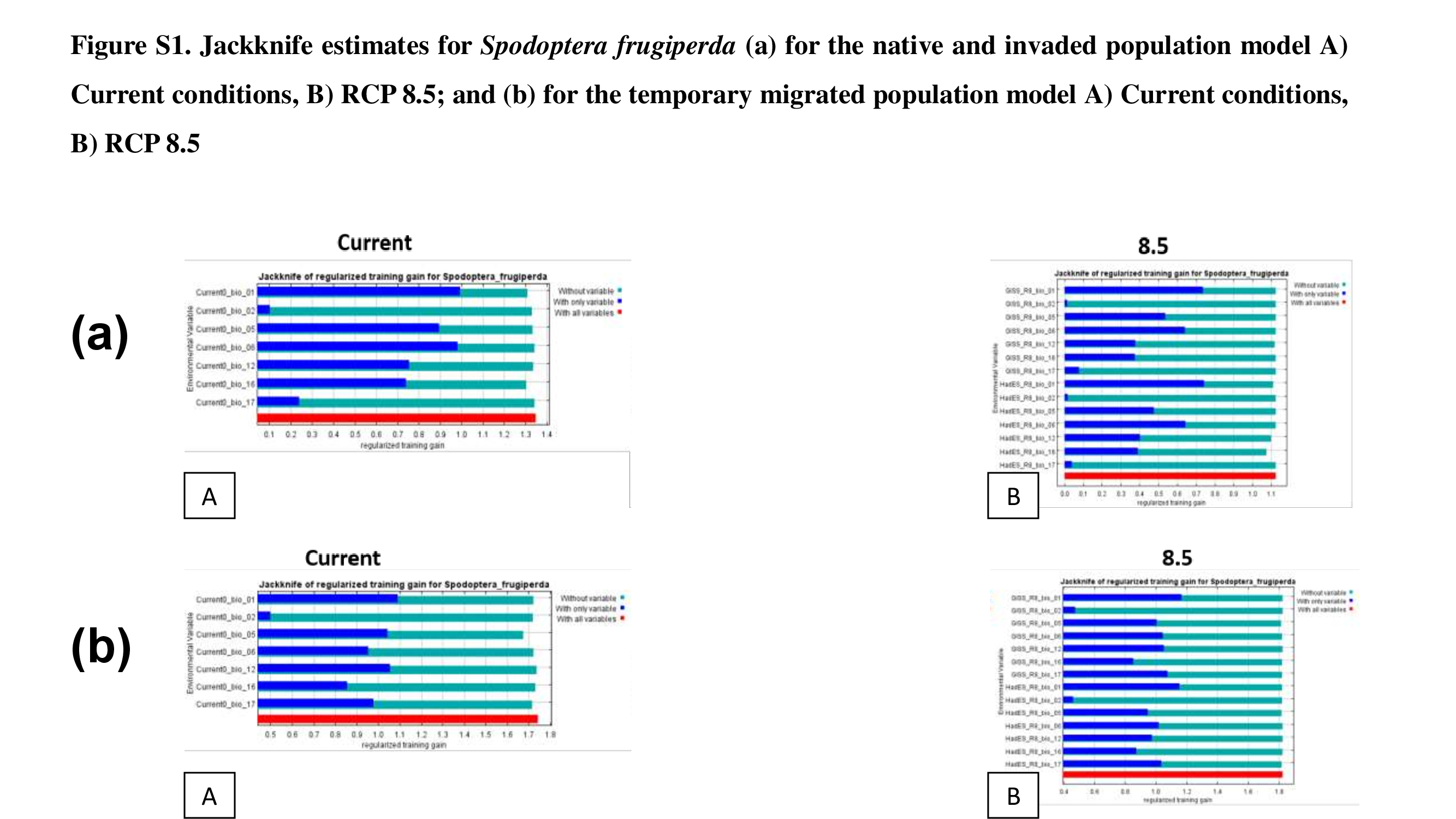

Supplement: Supplementary file 1 [file insects-12-00273-s001.zip › Supplementary materials/Figures S1-4/Figure S1.tiff]
